# Mapping the subjective importance of the topic ‘parenthood’ for parents with substance use disorder in inpatient rehabilitative care – an explorative qualitative study in Germany

**DOI:** 10.1186/s13011-026-00707-8

**Published:** 2026-02-07

**Authors:** Ananda Stullich, Jan Gehrmann, Johannes Stephan, Jana Dehner, Matthias Richter, Laura Hoffmann

**Affiliations:** 1 Chair of Social Determinants of Health, Department Health and Sport Sciences, TUM School of Medicine and Health, Technical University of Munich, Munich, Germany; 2 Department Clinical Medicine, Institute of General Practice and Health Services Research, TUM School of Medicine and Health, Technical University of Munich, Munich, Germany

**Keywords:** Addiction, Rehabilitation, Parent, Child, Children, Substance use disorder, Intervention, Qualitative research, Qualitative content analysis

## Abstract

**Background:**

In Germany, about 3 million children live with at least one parent with substance use disorder (SUD). Those parents face multiple challenges in daily life that concurrently affect their children. The inpatient stay of parents with SUD is seen as a possibility for an intervention (Kontext-Sucht-Intervention), centering on parents’ requests. Therefore, this study focuses on the importance of the topic of parenthood and parents’ perceived needs in inpatient rehabilitative care in Germany.

**Methods:**

We conducted 37 semi-structured interviews with parents with SUD between March and September 2023 in seven inpatient rehabilitative clinics in Germany. Two clinics carried out the Kontext-Sucht Intervention, and we recruited five clinics without structured interventions on parenting to contrastingly explore the needs and derive the meaning of the topic from the parent’s perspective. We analysed the interviews using content-structured qualitative content analysis.

**Result:**

The results show that the topic parenthood is important to parents in inpatient rehabilitative treatment, as they recognise the influence of their disease on the upbringing of their children. Parents identify a gap in the support system, wish for a positive future for their children, and therefore address several parenting-related topics for treatment: e.g., setting boundaries, integration of accompanying children, partnership, single parenting, siblings, and reduction of exhaustion resulting from parenting tasks. They also citicise the lack of specific, structured therapeutic interventions for parents.

**Conclusion:**

The findings suggest that there is a need for a parenting-focused intervention in inpatient rehabilitation, which should be provided by the rehabilitation system. The results are in line with general findings on families and substance use disorder purposes in SUD care.

**Trial registration:**

DRKS00030950, (Registration Date 25.01.2023).

**Supplementary Information:**

The online version contains supplementary material available at 10.1186/s13011-026-00707-8.

## Background

The latest data on substance use in Germany indicate that, in 2021, 84.7% of the adult population consumed alcohol, and 9.3% used any illicit drugs [1]. A total of 1.6 million people in Germany are dependent on alcohol [2], and 8.2 million people are dependent on (il-)legal substances or gambling [3]. The misuse of (il-)legal drugs and associated substance use disorder (SUD) causes various problems for those using them, society, and their families [3, 4]. SUD provokes direct health care costs, including for treatment, associated crime, incapacity to work, early retirement, and mortality [5, 6]. In Germany, about 3 million children are living with at least one parent misusing any illegal drug or alcohol, though this is likely an underestimate due to unreported cases [7]. Another study based on German Epidemiological Survey of Substance Abuse (ESA) from 2018 thus estimates the percentage of minors living with a parent with SUD to lie between 11.2% and 20.2% [8].

### Impact of SUD on parenting

Several studies indicate that parents with SUD have difficulties raising and fostering their children [7, 9]. Parents can be mentally or physically absent during their consumption or withdrawal phase [4, 10], and other studies report on child neglect or abuse [4, 9, 11]. Many of the arising health difficulties are related to SUD (e.g., acquisition crime) and the social context (e.g., “unhealthy” friends and partners) [4, 10, 12]. The circumstances (physical, mental, environmental) under which children are raised can result in physical and mental diseases [7, 13, 14].

The everyday challenges parents face within their parental role and duties can lead to overwhelming mental states, which are one of the most significant relapse factors [15]. Overwhelming mental states can, for example, arise from crying children, full-time schedules, fewer possibilities for healthy compensation techniques (e.g., exercising while caring for a three-year-old), and conflicts in daily routines with children depending on their developmental state and age. Hence, it is crucial to prevent these overwhelming mental states, as they may contribute to relapse or adversely affect children’s health.

Previous studies indicate that the topic of ‘parenthood’, which includes all parental duties, parental roles and responsibilities, parent-child bonds, etc., needs to be reflected within SUD-focused medical treatment [16, 17]. In Germany, parents with SUD can be treated in inpatient rehabilitative facilities with or without bringing their children as ‘accompanying children’ (until the age of 12). Usually, the parents stay in the clinics for up to 24 weeks, depending on their misuse of substances and the disease severity [18]. This lengthy treatment period provides a unique opportunity to integrate a structured programme on parenting-related aspects.

However, evaluated parenting programmes in Germany are currently only available in outpatient settings, including the parenting programme SHIFT [19] and a modularised programme to strengthen the resources of parents with SUD or psychiatric diseases [20]. In Ireland, the Parents under Pressure programme was successfully transferred from an in-house individual setting to a group setting in residential care for parents with SUD [21]. To our knowledge, there are no evaluated, structured parenting programmes to support parents with SUD in inpatient rehabilitative care in Germany. To close the gap of missing interventions in inpatient rehabilitation in Germany, the model project “KontextSucht” - “AddictionContext” develops, implements, and evaluates an intervention (Kontext-Sucht-Intervention = KSI). The project’s objective is to develop a needs-oriented KSI, focusing on parenting behaviour, parenting skills, parent-child-bond, drug abstinence, and employment of parents with SUD.

In order to better understand the role of parenthood for parents with SUD in inpatient rehabilitation, it is important to generally understand the parents’ perspective in detail. Little is known about the importance of the topic of parenthood and the subjective needs of parents with SUD in inpatient rehabilitation in Germany. To close this gap, our study aims to answer the following questions:

1. In what ways is the topic of ‘parenthood’ important to parents in inpatient rehabilitation in Germany?
2. What are the needs of parents in inpatient rehabilitative care in Germany?

## Methods

This paper is based on an analysis of data collected in the project “KontextSucht”, which uses a mixed-methods design with a feasibility study (stage one) and a benefit assessment (stage two) [22]. It is part of the funding line ‘rehapro’ by the German Federal Ministry of Labour and Social Affairs (Bundesministerium für Arbeit und Soziales). The German pension insurance of Central Germany (Deutsche Rentenversicherung Mitteldeutschland) leads the overall project. Two rehabilitation clinics, the MEDIAN Klinik Römhild and Barbarossa-Klinik Kelbra, developed and executed the KSI. The Ethical Review Committee at the Technical University of Munich approved the study (reference number: 2022-­624 s-­KH).

This paper focuses on the qualitative data collected during the feasibility study that aimed to explore all relevant aspects to develop a needs-oriented intervention. As part of this broader aim, we explored the importance of the topic ‘parenthood’, and the subjective needs for parents with SUD in rehabilitative care in German inpatient rehabilitation clinics. In addition, it aims to investigate parents‘ views on developing and implementing an intervention on the topic of parenthood more generally. The results will influence the revision of the KSI after the feasibility study.

### Settings and intervention

During the feasibility stage of the project (March 2023 – April 2024), the staff of the two intervention clinics first developed an innovative intervention for parents with SUD (KSI) and then implemented it. We collected qualitative data from both intervention clinics (clinics with accompanying children), as well as five additional clinics (exploratory clinics, three with accompanying children, two without accompanying children) to assess the needs of parents treated in rehabilitative care in Germany. Exploratory clinics had no structured programs or interventions on parenting during inpatient rehabilitation. Including both types of clinics was essential to gain a comprehensive understanding of the relevance of this topic within the German rehabilitation context more broadly. The evaluated KSI is intended to be transferable to other inpatient rehabilitation clinics in Germany. Therefore, it was crucial to explore not only the perceived needs and importance of the topic in the intervention clinics – where considerable attention has already been devoted to this issue – but also in rehabilitation clinics without a dedicated focus and evaluated programme addressing the topic. We focus on the patients’ perspective and their position as parents with SUD in long-term treatment for ceasing substance use of 12 to 24 weeks, which is free of charge for patients.

### Preparing and conducting the interviews

Study participants were recruited between January and September 2023 by the clinic staff, who informed them about the study.

The inclusion criteria for participation were:

- Parents with at least one child under the age of 14 years.
- Parents within the inpatient rehabilitative care for SUD.
- Fluent in German.
- Voluntary participation and signed informed consent.

The staff used a print leaflet and handed it out to the study participants. If participants agreed to take part in the interviews, the staff scheduled the interviews in consultation with the responsible researcher AS. AS conducted the semi-structured interviews face-to-face in the clinics, in a quiet, suitable room. Before the interviews, participants read an information sheet and AS answered all potential questions. After this, participants signed a consent form. Incentives (vouchers for a shared experience with a child with a total value of 20 euros) were distributed by raffle.

The interview guide was developed using Hellferich’s principle for developing a structured interview guide with a clear methodological approach [23, 24]. AS and LH critically reviewed all questions and adjusted them after the first interviews. Some questions were created in simple language, as SUD can lead to cognitive problems [25]. The final version of the interview guide covers the following aspects, depending on the intervention and exploratory clinics: Subjective perspectives on parenting, subjective problems in parenting, needs for support on parenting during treatment, knowledge about support systems for parenting issues, general living situation, therapeutical options on parenting skills, parent-child-bond and integration of the children during treatment, fulfilment of needs/demands and wishes concerning parenting aspects and treatment (see Additional file 1).

We created pseudonyms using four standardised questions that generated a code that can only be reproduced with the participants. All interviews were recorded using Olympus WS-853™ digital voice recorder and stored in password-protected digital devices and servers of the Technical University of Munich.

### Description of participants

This analysis included 37 interviews with rehabilitants in the intervention clinics (*n* = 16) and exploratory clinics (*n* = 21). All interviews were conducted between March and September 2023 and lasted on average 28 min (Min = 13; Max = 70). We included all participants who expressed interest during the recruitment period, and continued recruitment until theoretical saturation was reached. The interviews were transcribed and anonymised by a professional transcription service that is familiar with scientific transcriptions using a verbatim system [26]. All interviews were conducted, transcribed, and analysed in German. For this publication, quotes were translated into English by AS and critically reviewed by LH, JG, JS. All authors are native Germans, with English as their second language.

### Sample characteristics

Of the 37 study participants, 57% described themselves as female. They were, on average, 35.59 (SD: 6.06; Min = 19; Max = 45) years old. Overall, 56.75% consumed more than one substance. They had on average 1.89 children, and their children were, on average, 7.03 years old. 35% of the parents reported having at least one child with them in the clinic. All study participants were German citizens. No interview was cancelled or withdrawn at the point of publishing this study.

A differentiated description of the sample characteristics can be seen in Table 1:

**Table 1:** Sample characteristics

	Intervention clinics (*n* = 16)	Exploratory clinics (*n* = 21)	∑ (*n* = 37)
**Gender**	16	21	
Female	10	11	21
Male	6	10	16
**Age (years) (mean/SD)**	35.25/5.43	35.86/6.62	35.59/6.06
Min	22	19	19
Max	42	45	45
**Number of children per parent (mean/SD)**	1.81/1.05	1.95/0.80	1.89/0.91
Min	1	1	1
Max	5	4	5
**Age of children (mean/SD)**	6.43/4.23	7.43/4.97	7.03/4.68
Min	1	< 1	< 1
Max	19	18	19
**Parents**			
With accompanying child	7	6	13
Without accompanying child	9	15	24

Note. Min (minimum), Max (maximum), SD (standard deviation)

### Data analysis

We used qualitative content analysis to analyse the interview data in a rule-based way [27, 28]. We utilised a content-structured form of deductive-inductive qualitative content analysis [29, 30], developing main categories deductively and creating subcategories inductively [29–31]. The analysis was conducted using MAXQDA Analytics Pro 2022 (release 22.8.0)™.

We formulated initial deductive main categories using the main topics of the interview guide for an preliminary structure [29]. Due to the heterogeneity of the data, we continued inductive coding to cover all relevant aspects/themes. Inductive codes were sorted and organised into broader subcategories while simultaneously writing precise descriptions of the categories. These categories were reviewed after approximately 30% of the data had been coded. We sorted inductive codes that did not fulfil the criteria of the deductively predefined main categories separately for the analysis. The first author analysed the complete data, while LH and JD coded several interviews separately. Afterwards, codes and categories were discussed until all coders agreed on the codes and categories, and revisions were made accordingly (see Additional file 2). First interpretations of the data were carried out separately by AS, LH, and JD. We performed final interpretations and analysis iteratively in several group discussions (AS, LH, JD, JS, JG), including a discussion on quotes, and when saturation was reached. All researchers took an outside perspective on the data material, as none of them are parents with SUD in rehabilitative care. Researchers followed the *COnsolidated criteria for REporting Qualitative research (COREQ*) guidelines for reporting qualitative research [32] (see Additional file 3).

## Results

Throughout this section, we first outline the relevance and importance of ‘parenthood’ in inpatient rehabilitative care. Then, we present the needs of the respective parents in inpatient treatment. We present the (sub-)categories of the analysis in Additional file 2. The description of the quotes includes information on gender (m = male; f = female), the age range of the participants in years, and if the interviews were conducted either in the intervention clinic (IC) or exploratory clinic (EC).

### Importance of parenting in inpatient rehabilitation clinics

The parents presented parenthood as a major reason for treatment and reflected on the negative impact of SUD on their family and children.

### Parenthood as a motivation for treatment

Parents in rehabilitative SUD treatment describe their role as a parent and parenting as a significant topic for their choice of treatment. In some cases, being a parent and having children is indicated as the only motivation for treatment. Participants reflect on their own biographies, particularly their childhood experiences of being parented, and translate these experiences into aspirations for their children’s upbringing. The great importance of the topic of ‘parenthood’ during treatment is seen in parenting, family cohesion, and interaction, or parenting skills by the parents. The interviewed parents wish for a positive future for their children. Further, they wish not to transfer their own negative experiences to them. Parenthood and the symptoms of SUD cannot always be considered totally disjunct, which is illustrated by the following quote of a patient who sees his daughters only on weekends.

*That was the path that led me into depression*,* no longer having them around me every day; also*,* always having to say goodbye to the girls on Sunday*,* that was the time here when I took [drugs] most often […]. (m; 25–29; EC)*

The interviewed parents often present a dichotomous decision between either continuing the drug use or ceasing it to keep their children, as both *“drugs and kids do not work together” (m; 40–44; EC).*

*Does not work. Doesn’t work. The most important thing*,* the children in that sense*,* is neglected. So*,* you tell yourself it’s working*,* everything is great and/or it’s not. So*,* the house of cards collapses quickly and the children fall by the wayside in any case. (f; 30–34; IC)*

Explicitly the “fall by the wayside” can be summarised to the following aspects mentioned in the interviews such as hiding SUD, not being able to support their children age-appropriately (e.g., with school-related tasks like homework, parent-teacher-conferences, early childhood development support, speech therapy) or keeping up daily structure (e.g., pick-up and drop off service, routines in eating and sleep). This leads to limited everyday ability and problematising one’s own drug consumption while not completely fulfilling the role of a parent.

### Perceived negative impact of substance use on children

Participants also reflected (as part of their inpatient therapy) on the consequences for their children. They mentioned a variety of physical and mental health consequences, including behavioural problems, co-dependency, parentification, and foetal alcohol syndrome.

*And what I only became aware of here is that my children often ask me*,* or like to ask me*,* if they can open a beer for me*,* which previously made me a bit/ Well before I was here [clinic]*,* I just smiled about it*,* but since I’ve been here*,* I just find it sad*,* the behaviour*,* isn’t it? And it also shows how much the children are used to it. I’ll just handle it differently hereafter. (m; 25–29; EC)*

As a result of this, the interviewed parents want reclaim responsibility in three dimensions that all seem relevant for their capacity to fulfil parental duties in everyday life: First, they want to take responsibility for themselves; second, for their children; and third, for their social context, including work and other relatives and family members.

### Addressing parenting during treatment

Parents see their treatment as a chance to regain the ability to fulfil these responsibilities but address certain needs during the therapeutical process. By contrast, some parents reflect that they explicitly do not want to focus on parenthood:

*I think it’s fine the way it is. […] There used to be an approach here that you have a mother/ You have a group here*,* a reference group*,* and there was a therapist here*,* a therapist*,* who wanted to organise ONLY a mother’s group/ But the mothers don’t want that either*,* because then it’s only about children. But we’re here because of us*,* right? (f; 30–34; EC)*

Some parents perceive it as a hurdle to focus too much on parenting aspects, and they may risk losing focus on themselves and their recovery of SUD. Parents with accompanying children generally describe the time in the rehabilitation clinic as an intense time together with their children: *“You are also intensively involved here with*,* with your child*,* day in*,* day out.” (f; 30–34; EC)* The parents interviewed for this study would like to be supported in coping with issues during their inpatient rehabilitation stay because they firmly believe that their stability and ability to remain abstinent depends on how productively they deal with these challenges. Irrespective of whether the clinic addressed some of the presented needs beforehand, parents explain that they profit from the other patients in non-structured peer-to-peer discussions during their stay. Even in cases where interviewees are dissatisfied with the general conditions for parents in the clinic, they highlight the high value of harmony through fellow patients and peer-to-peer support. One parent summarizes the overall process during treatment as follows:

*Let me cut a long story short: children do well when they have healthy*,* stable parents. This means that everything that helps the parents in principle ultimately also helps the children. (f; 40–44; IC)*

### Needs of parents with substance use disorder

Parents reflect on the consequences for their children but are not always capable of changing circumstances or personal behaviour, such as frequent and quick mood changes and missing emotional control, resulting in inconsistent and unpredictable behaviour towards their children.

*On the one hand*,* I’m just growing into this role [parental role]. And (…) on the other hand*,* it’s also the case that the children are already*,* I don’t want to say “badly disturbed”*,* but you can already see a behavioural disorder*,* can’t you? In many of them. And (…) the fact that we addicts have to deal with ourselves first and then look after the children at the same time makes it twice as difficult somehow. […] I think addicts need a lot of support. (m; 35–39; IC)*

This patient with two children describes developing and refining his role as a parent and adequately meeting both his own and his children’s needs as challenging and equally in need of support. Furthermore, parents address that it is difficult to reflect, explain, and control the emotional and physical needs depending on the child’s age: *“Because I didn’t know yet, how to help my child with the emotions. How I should convey [the emotions].” (f; 30–34; IC).* Consequently, they want to discover and learn about their own needs and how to deal with emotions. Recognising one’s own emotions and caring for personal needs provides the foundation for meeting the child’s needs.

Parents report feelings of excessive demands in parenting situations up to being overwhelmed. Going into more detail, the participants relate to the topic of parental discipline, including enforcing family rules and boundaries. Sometimes, they report practising inadequat parenting behaviour.

*And sometimes I think that we addicts are often overwhelmed by situations*,* especially IF you’ve been through so much in childhood and bad things that you might not often be able to react the way you’d like to because it’s just not given to you at that moment*,* so to speak*,* and that’s what we learn here. (f; 35–39; EC)*

This aspect is often mentioned in relation to the desire to be a ‘good’ parent who is ‘present’ for their child in every situation. It is closely related to the parental role, which is described as not fully fulfilled in the active consumption phase, where the focus is mainly on consumption, withdrawal, etc.

### Gaps and resulting needs in support systems for parents with substance use disorder

In situations of excessive demands, parents may use certain support options. Some parents report that they have never received any form of support. In cases when parents are given support, they receive private and/or professional support. They get help from their immediate social context (family members and close friends). Professional support is used in the forms of family assistance, counselling centres for people with SUD, youth welfare offices, educational counselling centres and outpatient psychotherapy/clinics.

*In difficult situations*,* for example*,* when my child is being taken away from me. Or*,* for example*,* what I can do if my child is too fidgety. Or different situations*,* if/whether my child is healthy or what I can perhaps improve. (f; 30–34; EC)*

If there is a threat of placing children in foster care and removing custody, parents make use of several different support systems at a time. Support with the application to the authorities (e.g., health insurance, youth welfare offices, physicians, educational institutions) is an important aspect as bureaucracy in Germany is a hurdle.

The given explanations in the following quote refer to the mental and physical health issues of the child, behavioural challenges, and if parents feel the need for educational advice during treatment.

*Yes*,* it’s how I bring up my child properly. And*,* because I sometimes see mothers walking around with a pram and a beer in their hand. I don’t want to do that. (f; 15–19; EC)*

Educational advice also includes setting boundaries with the child or how to improve communication, if insecurities in parental duties are present.

*I should set a limit for her birthday and stuff and so*,* because I’ve always bought her everything. […] As a father you just do that*,* I don’t know. And that’s just*,* I didn’t draw the boundaries and that’s what I have to do. And she [the therapist] says*,* she told us directly*,* that I should set more boundaries and then say “no” sometimes*,* and somehow that’s better because otherwise*,* she’s walking all over somebody*,* the daughter. (m; 35–39, EC)*

Setting adequate boundaries as part of being a caring parent and avoiding conflict is part of the learning process for this father during treatment.

Not all parents can confidently use existing in- and outpatient professional support systems, or the existing support is not seen as sufficiently addressing their needs. This leads to several unmet needs. These can be differentiated into personal and structural needs that are not always directly related to the inpatient treatment. On a personal level, the participants want to learn assertiveness, stress reduction, take responsibility, and redefine or even discover their parental role. Structural aspects inside the inpatient rehabilitation clinics include treatment itself and environmental structures. The participants often criticised that the treatment is not sufficiently systemically focused towards their situation as parents and evidence-based concepts for parenting with SUD are unavailable in the respective clinic. Therapeutic sessions with the children, the parent, and the therapists are considered beneficial but extremely rare. Parents also discuss the need for counselling regarding concrete parenting skills in everyday tasks (e.g., brushing teeth, taking meals, bringing children to bed, and separating before children go into childcare) inside the clinical setting.

### Needs within the integration of children in inpatient care

In Germany, the possibility of bringing accompanying children (< 12 years) into some rehabilitation clinic leads to several specific needs in relation to them. As children can be between 0 and 12 years old, they have different needs regarding furniture and safety, as well as toys. This includes accompanying children and children on visits. Parents with children on visits further discuss that they feel like they have lost connection/contact with their child, which, after that 12- to 24-week stay, needs to be rebuilt. This is framed as an aspect of alienation. They feel the need for maintenance interventions. When children are partly living outside the rehabilitation clinic, parents wish for the possibility of weekend or holiday stays for their children in the facility to keep contact and not lose the parent-child bond. This could improve their relationship within a safe environment, where problems can immediately be addressed by professionals.

House rules, in general, are discussed critically. Parents mark that more visiting times of children in the clinic or trips home could be beneficial, but the institution is mainly enforcing rules that focus on patient treatment for people with SUD, completely neglecting the consequences of such a long interruption of immediate parent-child interaction. In addition, parents like to prepare their children’s favourite meals, with fresh food. However, the equipment or free usage of kitchens and bringing perishable food from outside is restricted in some clinics. This leads to dissatisfaction with the diversity and quality of food that parents can provide for a relatively long stay between 12 and 24 weeks.

*I’m not happy with the cheese*,* […] we’re not allowed to cook and freeze food for the child ourselves*,* for hygiene reasons. (…) […] That really annoys me. I always cooked my own food at home. He got so many vegetables*,* and I also made sure they were organic*,* and he got something different every day. (…) And then you’re not even allowed to bring your own food from outside. That means he gets bananas every day. He gets cucumber every day. […] What else can you give him? (…) […] Kiwi okay yes*,* but they don’t pay attention to organic quality here either*,* no. My family nurse really emphasised that to me. I’m always really careful about that. (m; 20–24; IC)*

Rules in the clinic concerning free movement and the duty of supervision for ‘older children’ (according to our analysis, starting at school age, so children are five to six years of age) can be challenging, as *“the children are NOT allowed to leave the centre alone and go back to their shared room*,* whether they go to kindergarten or the after-school care centre*,* but must be picked up.” (f; 40–44; EC)* This is seen as a developmental restriction on gaining independence from their parent. The parents explain, that children are becoming increasingly independent of their parents is part of growing up and is inhibited by the clinic rules.

Older accompanying children for example

*aren’t allowed in the fitness room: the older children have nothing to play with here. Of course*,* there’s a small playground*,* but she’s twelve now. She doesn’t go to the playground anymore*,* does she? If she WANTS to go out*,* because she now has one or two friends outside at school*,* right? She can meet up with them in the afternoons. She’ll have to ask my therapist.(m; 35–39; IC)*

This quote also shows that the child needs to ask the father’s therapist to go out to meet friends. So, the father’s power to make educational decisions for their daughter is externally determined by the clinic’s staff. In this case, the clinic equipment that can be used by patients can not be used by children for leisure activities. The father adds that one time when a joint sports programme was scheduled for father and child, the child was only able to participate during school holidays, due to overlapping time schedules of clinic and school that made it impossible for school children to participate. Therefore, the parents demand more flexibility and options for older children’s leisure time activities.

Regarding the integration of children in therapeutical work, participants emphasise offers as insufficient or, in some cases in the exploration clinics, as non-existent.

*Well*,* it’s a bit worse here*,* because there really aren’t any*,* not for mother and child therapies or anything. It’s really just for you alone. There’s therapy every two weeks*,* which lasts an hour and a half. We do arts and crafts and paint*,* don’t we? So*,* there aren’t many programmes with mother and child together. Unfortunately. (f; 30–34; EC)*

As can be seen in this quote, the mother describes the need for therapies together with their child to work on parent-child-relationship tasks. Others add that they miss single treatment for their children to process what they have experienced and to understand their parents’ illness. Whenever rehabilitation clinics offer special therapeutical groups (leisure time activities, cooking groups, creative groups, sports groups) where parents and children can participate together, this is seen as profitable and invariably positive. Parents describe the exchange and feedback on parenting aspects with the personnel of the childcare units, which are organised individually by each clinic. Parents can sometimes make appointments with the staff of the respective childcare facilities/schools.

Parents also see limitations in their options to support their children during their clinical stay. Support such as personal tutoring for school is not as straightforward as at home. Parents, moreover, wish for relief services, such as babysitters, which are not allowed during clinical stays. The fact that rehabilitation care focuses on adults (in this case, the parents), while their children are seen only as accompanying children and not as patients themselves, results in therapeutical offers that only address adults. This is why the participants feel a strong need for leisure activities for children, quality time, excursions, and events like family days.

### Specific treatment requirements

We asked all parents (with and without accompanying children) directly about their needs of which aspects about parenthood should be included as specific topics in their rehabilitation treatment. Three topics will be explained in detail: partnership, single parenting, and siblings.

Partnership, or ‘the other biological parent‘, is important to all, whether they still live together as a couple, are separated, or live in other relationship configurations. In every case, parents or partners must coordinate the care of their child. Both partners can practice different parenting styles, leading to conflicts and irritation for the children. Conflicts in partnership (including child custody disputes), in general, are a crucial problem to be solved. Conflicts can appear in separated partners as well as in current partnerships and are considered challenging and negatively affecting the children by the interviewed parents. If parents and children are separated, this can lead to difficulties in building a stable parent-child bond.

*[…] because I am currently separating or have separated from the father. And because (…) there is the stress that you have with a child/ I’ll be a single parent in the future*,* which you also have at home*,* so it wouldn’t help if I reorganised my life here [in the clinic] WITHOUT a child and then came home and suddenly had a child. Yes. (f; 30–34; EC)*

The mother believes that she has to adapt to the new situation after separation from her partner, together with her child and all aspects and challenges that are related to the new situation of being a single mum.

Separated partnerships lead to single parenting in at least one of the parents. If the interviewed parent is a single parent, they describe parenting as highly demanding.

*Especially with a child and being a single parent*,* which I am*,* but which I don’t really mind*,* it then spills over in such a way that I then reach far too readily for amphetamines*,* which give me structure*,* which make me clear*,* which make me clearer. (f; 40–44; EC)*

It can also be seen that some parents use their drug to structure themselves in their daily routines. Moreover, being a single parent means being responsible for everything at any time. This mother illustrated what it means to a parent with SUD in everyday life:

*Sometimes you no longer have the drive to do activities with your child. You don’t have the patience to listen. In the morning*,* when the child starts to talk (imitates “babbling”)*,* who else is she supposed to talk to if only mum is there? If that then becomes too MUCH and you actually need time for yourself first to wake up and get along. It had nothing to do with morning grouchiness*,* but simply with a nervous exhaustion that is simply no longer there*,* yes. And maybe sleeping for a long time too*,* because being tired because of being on it [the drug] means you don’t get enough sleep or any sleep at all. (f; 40–44; EC)*

The question of sleep as a resource for parenting is highlighted and withdrawal states are a particular challenge for single carers as they are the only reference person for the child. This puts pressure on the family situation, and results in the need to be able or to learn how to handle those situations.

Separation is present not only between the parents but also between siblings.

*Or bringing siblings together*,* that was a big problem for us. My daughter had no relationship with her brother. That’s improved here*,* definitely*,* but there’s also this sibling bonding. There are three of us. But also this*,* yes*,* this sibling bond. There’s just nothing [therapeutic offer] here [clinic]*,* for example. That’s also important*,* isn’t it? Because I’m still involved in that [sibling bonding process]*,* in addition*,* and that’s exhausting for me too*,* yes? (f; 40–44; EC)*

This quote shows a completely different requirement for parents, that is rated as ‘exhausting’: The parents’ task to mediate between their children, where this interviewee wishes for professional support.

## Discussion

This qualitative study within the inpatient rehabilitative treatment for parents with SUD is among the first studies in Germany outlining the importance of the topic of ‘parenthood’ for the treatment of parents with and without their accompanying children in Germany. This topic is highly important for parents with SUD in inpatient rehabilitative treatment, where they lack access to structured therapeutical offers. Parents critically reflect the consequences of their SUD on the upbringing of their children. Several topics are described to be important, including partnership, single parenting, and siblings. Moreover, parents want to learn to better interpret their children’s emotions, set age-adequate boundaries, and reduce exhaustion as a result of overwhelming parental tasks in everyday life. During the revision of the KSI after the feasibility study, a specific focus is set on these topics. Integrating accompanying children in everyday hospital life seems improvable.

Reflecting the negative effects of the unfulfilled parental duties in the past, the parents clearly state that they want a positive future for themselves and their children, as has been outlined for parents in other contexts [33, 34]. This includes age-appropriate parental behaviour in a state of abstinence in which they can react appropriately to conflicts. Previous literature indicates that, if those parental conflicts are carried out in a dysfunctional manner, they can have negative effects on children and need to be minimised [35, 36].

Parents in our study want to fulfil their parental role positively and (re-)fulfil the complex responsibilities placed on them, including controlling mood swings. A literature review on the impact of parental SUD on children also marks the negative effects of inconsistent, disengaged or harsh parenting practices on children [37]. It is also relevant for the interviewed parents to be able to assess and satisfy their own needs and the needs of their children appropriately, interrupting the inter-generational cycle of SUD, which was analysed by previous studies [4, 7, 38].

The wish for systemic treatment approaches (e.g., including treatment sessions for children, relatives) for parents in psychotherapeutic care is well known, and multiple approaches exist [39–41]. The professional feedback on parental behaviour is seen as vital, which is supported by an experimental study by Shanley and Niec that concludes that parents generally profit from in-vivo feedback on parent-child interactions [44].

A wide variety of separation experiences play a role. The negative effects of the separation of two parents on children are also widely known in other contexts [42]. The separation of siblings is not only problematised in the context of SUD but also linked to various challenges for the parents [43, 44]. Rebuilding lost parent-child relationships is a priority identified in this study. The single parents in the study, just like other single parents without an illness, are faced with juggling various responsibilities concerning their children simultaneously, corresponding to a broad scientific consensus [45, 46].

The situation in the clinics is described as challenging, as house rules restrict flexibility and make it difficult to establish and maintain contact with important caregivers living outside the clinic due to, for example, restrictive visiting rules. This is in line with other studies focusing on children visiting their parents [41, 47]. It is not surprising that in clinics where only adults are treated, children only have the status of accompanying children [18] and are, therefore, only supervised, and not therapeutically addressed themselves, leading to considerable challenges for parents. This also applies to the limited possibilities of age-appropriate support for accompanying children, as parents are not as self-determined and flexible as outside the clinic.

Parents used private and professional support systems in all areas they identify as problematic. Parents strongly criticise that there are few systemic approaches in Germany that include support services for the children and other family members, which is in line with previous findings from Bröning et al., who criticise the lack of systematic, homogeneous evidence-based programmes for children [48]. In the German ambulatory setting, some interventions focus on this topic, e.g., the TRAMPOLIN intervention [49] or the SHIFT programme [19, 50, 51].

### Strengths and limitations

This study includes 37 semi-structured interviews with parents from seven rehabilitation clinics that put our results on a strong empirical base. Studies on the group of parents with SUD with and without accompanying children in inpatient settings remain rare; this study, therefore, makes a valuable contribution to the research field. Even more men than women with SUD are treated in 2023 in inpatient care in Germany [52]. The sample of this study reflects an almost equal gender ratio. The limitations of the study include setting specific restrictions, such as focusing only on inpatient, rehabilitative treatment in Germany. Possibly, the experiences and needs of outpatient treatment are different. This study focuses specifically on long-term treatment for ceasing substance use and not on detoxification treatment. The individual situations in inpatient rehabilitation clinics and family situations are highly heterogeneous, and we recommend taking these results as guidance but individually looking at each situation in practice. All study participants were German citizens, natively speaking German and White, so no migration aspects or language barriers could be considered. Participants were selected by the clinical staff. The study was based on self-report. Social desirability bias is possibly influencing data as parenting, and substance abuse are highly stigmatised and sensitive topics. Translation of quotes from German to English can be a limitation as exact word meanings cannot be guaranteed.

## Conclusions

This study answered the questions on needs and the meaning of the topic of ‘parenthood’ in rehabilitative care. We conclude that the topic is highly valuable, and a wide variety of personal and structural needs are present. Participants from the explorative as well as the intervention clinics show a great need for the topic, irrespective of whether the clinic offers the opportunity to bring children to the facility or not. This can be seen as an indicator to address the topic, as parents stay parents, no matter if they have their children with them or not. The presented results should be recognised when developing and adapting the KSI or other parenting programmes in inpatient rehabilitative care. This study should be seen as a strong plaidoyer for a structured, evaluated programme in inpatient rehabilitative care in Germany, as the KontextSucht project progresses.

## Supplementary Information

Below is the link to the electronic supplementary material.

Supplementary Material 1

Supplementary Material 2

Supplementary Material 3

## Data Availability

Not applicable.

## Declarations

### AI tools

AI tools (DeepL, Grammarly) were exclusively used for grammar, spelling corrections and translation accuracy. No AI tools were used for text creation or data analysis. After using these tools, the authors reviewed and edited the content as needed and took full responsibility for the publication’s content.

## Abbreviations

EX: Exploratory Clinic
F: Female
IC: Intervention Clinic
KSI: KontextSucht Intervention (AddictionContext Intervention)
M: Male
SUD: Substance Use Disorder
