# Supplementary material for: Mapping the subjective importance of the topic ‘parenthood’ for parents with substance use disorder in inpatient rehabilitative care – an explorative qualitative study in Germany
Source: Subst Abuse Treat Prev Policy. 2026 Feb 7;21:21. doi: 10.1186/s13011-026-00707-8 (PMC12977838; doi:10.1186/s13011-026-00707-8)
Supplement: Supplementary file 1 — Supplementary Material 1 [file 13011_2026_707_MOESM1_ESM.docx]

Interview Guide Intervention & Exploratory Clinic

In advance:

1. Consent forms
2. Remarks on the interview process
3. Creating the pseudonym
4. Transfer of the routing slip of the intervention units
5. Information on the start of the recording

| **Topic** | | **Questions (translated)** | **Easy language (not translated)** |
| --- | --- | --- | --- |
| **1** | **General information** | When were you born? |  |
|  |  | Which gender do you feel you belong to? | Welches Geschlecht haben Sie? |
|  |  | How old is/are your child/children? |  |
|  |  | Do you live in the city or in the countryside? |  |
|  |  | Please tell me if and in what form you currently work when you are not in this rehabilitation centre. | Haben Sie aktuell Arbeit?  Was machen Sie in dieser Arbeit? |
|  | | | |
| **2** | **SUD** | What SUD disorder are you currently undergoing treatment for?  Who referred you to the rehabilitation centre? | Wegen was sind Sie hier? |
|  |  | Since when do you think you have been suffering from SUD? How do you determine this?  How many times have you undergone withdrawal treatments so far? | Seit wann meinen Sie, macht Ihnen der Konsum von Drogen/Alkohol Probleme?  Warum finden Sie das? |
|  | | | |
| **3** | **Parenthood** | Are any of your children in this treatment centre with you? | Ist eines Ihrer Kinder hier mit dabei? |
|  |  | Why did you bring your child to the treatment centre? | Warum haben Sie Ihr Kind dabei? |
|  |  | Why did your child stay at home?  Who is your child currently with and why is he/she with this/these person(s)? | Warum ist Ihr Kind zuhause? |
|  |  | How important is the topic of motherhood/fatherhood or family for you during your treatment in general? | Ist das Thema Mutter-/Vaterschaft oder Familie in Ihrer Entwöhnungsbehandlung für Sie ganz persönlich wichtig? Falls ja/nein, warum? |
|  |  | Please tell me how you personally deal with the issue of children and family in combination with your SUD? | Laufen die Dinge eher gut, oder gibt es da auch Probleme bei der Erziehung/dem Umgang mit Ihrem Kind? |
|  |  | I would now like to ask you to think about the various support options for parents with SUD.  What support options do you personally know of for raising your children? |  |
|  | | | |
| **4** | **Life**  **situation** | Please tell me about the current living situation for you and your child(ren) outside this rehabilitation centre. |  |
|  |  | Who do you currently live with?  What is your current housing situation? |  |
|  | | | |
| **5** | **Treatment** | What aspects of your treatment at this rehabilitation centre affect you as a parent? | Welche Therapien fallen Ihnen ein, die etwas mit Ihnen als Mutter/Vater zu tun haben? |
|  |  | If you think about your treatment here: How do you work on how to educate and deal with your child so that everyone is well? | Können Sie mir erzählen, ob Sie mit einem* Therapeut*in daran arbeiten, wie Sie ihr Kind erziehen? |
|  |  | I want to ask you to tell me how you work with a therapist on your relationship with your child. |  |
|  |  | What opportunities exist to take advantage of therapeutic programmes with your child? | Würden Sie mir von den Therapien erzählen, bei denen Ihr Kind mit in der Therapie ist? |
|  | | | |
| **6** | **Modules of the KSI in detail (only intervention clincis)** | Questions focus on the details of the KSI modules (e.g., group size assessment) and are not used to assess the importance of the topic. |  |
|  | | | |
| **7** | **Needs/**  **concrete wishes** | In your opinion, what criticisms do you have of your treatment at this rehabilitation centre concerning the topic of family and children? | Was finden Sie schlecht, wenn Sie an Ihre Therapie mit Ihrem Kind denken? |
|  |  | When you think about parents, children, and family, what is going well during treatment? |  |
|  |  | Now, please think again about the aspects of your treatment that relate to you as a mother or father and your child/children:  Can you tell me what you personally would do differently? |  |
|  |  | Suppose you consider your opportunities to participate in treatment programmes with your child. How would you personally rate these programmes?  Are there certain things you wish for? | Wie finden Sie die Möglichkeiten mit Ihrem Kind zusammen hier eine Therapie zu machen?  Wünschen Sie sich hierfür noch etwas genaues? |
|  |  | Please conclude: How do you feel about your opportunities to learn on-site as a mother/father? |  |
|  | **Only included in intervention Clinics.** | I would now like you to describe to me what, from your perspective, can support/help you in your treatment to remain abstinent/clean/dry in the long term.  How do you feel about your ability to pursue gainful employment/work after your time in this rehabilitation centre? | Wie (gut) fühlen Sie sich vorbereitet, nach der Therapie wieder einer Arbeit nachzugehen? |
|  | | | |
| **8** | **General wishes** | Are there still things about your SUD and family that you want to talk about that we haven't had time to discuss yet? | Gibt es sonst noch etwas zu diesem Thema, das Sie mir gerne erzählen würden? |
