# Supplementary material for: Mapping the subjective importance of the topic ‘parenthood’ for parents with substance use disorder in inpatient rehabilitative care – an explorative qualitative study in Germany
Source: Subst Abuse Treat Prev Policy. 2026 Feb 7;21:21. doi: 10.1186/s13011-026-00707-8 (PMC12977838; doi:10.1186/s13011-026-00707-8)
Supplement: Supplementary file 2 — Supplementary Material 2 [file 13011_2026_707_MOESM2_ESM.docx]

(Sub-) Categories: Importance of the Topic “Parenthood”

| **Main Category (translated)** | **Subcategory (translated)** | **Explanation (translated)** |
| --- | --- | --- |
| Reasons for rehabilitation | Non-child-related | The motivation of parents to go to rehabilitation is coded. Particular attention is paid to voluntariness and what it does to motivation. e.g., Treatment because of the instructions of the youth welfare office or court, |
|  | Child-related |  |
| Reasons for SUD |  | Disease data and progression data for the topic of SUD are coded. e.g., When did the SUD begin? How does it manifest? How do parents define their SUD? e.g., craving, loss of functionality in everyday life, increase in consumption… |
| Referral to rehabilitation |  | The way in which the parents came to rehabilitation is coded. e.g., withdrawal clinic, pressure from youth welfare office, outpatient psychotherapist; if this is assessed or justified, it is also coded. |
| Reasons for the children's whereabouts |  | Why do the parents attend treatment with/without accompanying children? Where do the children stay? Evaluations of the decision are coded. e.g., the children remain in their familiar environment for social reasons, the grandparents look after them, and the children are in out-of-home care. |
| Importance of the topic of parenthood in treatment | High importance of motherhood/fatherhood in treatment | Code how parents define the importance of children and the topic of parenthood during treatment. |
|  | Parenthood as a secondary topic in treatment | Code if parenthood is not seen as a primary topic during treatment. |
|  | Responsibility | It is coded how the topic of responsibility is associated with the importance of parenthood in treatment. e.g., taking responsibility for one's own life, taking responsibility for children, taking responsibility for family and friends. |
|  | Abstinence | Code aspects, whereby the processing of the topic of parenthood is brought into a direct link with the parents' abstinence. e.g., child as support for abstinence motivation. |
| Dealing with family and SUD | Education of the child | It is about phenomenological reports of parents' personal “dealing” with the topic of SUD in the family context. The parents define "dealing" themselves. e.g., whether their children know/discuss the SUD, aspects of partnership and family, the role of the grandparents/friends, everyday descriptions of consumption and withdrawal, and the effects on family life, the parents and their children are included. |
|  | Contact with the child |  |
|  | Effects on the children |  |
|  | Partnership |  |
|  | Social context |  |
| Problematizing your own parenthood | Emotional problems | Everything that parents describe as problems is coded. These include a lack of knowledge about child development or educational skills, issues with setting boundaries, the children experiencing mood swings or other role conflicts, and neglecting daily duties. If parents describe the effects of circumstances and parental behaviour toward their children (e.g., retarded child development, parentification), this is coded. |
|  | Neglect of daily duties |  |
|  | Neglect of parental duties |  |
|  | Support for children |  |
|  | Effects of parental behaviour on the children |  |
|  | Lack of knowledge |  |
| Support options for parents | Knowledge of support options | Everything that the parents know or can imagine in terms of support options. |
|  | Support options used | Everything that the parents have used in terms of support options. |
|  | Normative attitude | This is about evaluations and attitudes towards different support options. The background to how these attitudes came about is coded. |
| General living situation (Domestic circumstances and contextual factors) |  | All aspects that are related to life, the living situation, etc., are corded. The interpretation of the term is left to the parents. It is also about the structural organization of everyday life. |
| Therapeutic aspects of parenthood |  | Specific treatment sessions that have something to do with the topic of family, motherhood, or fatherhood. |
| Therapeutic aspects of parenting skills |  | Therapeutic sessions that are related to parenting skills are summarized, and an attempt is made to capture situations in which this topic is presented. |
| Therapeutic aspects of the child relationship |  | This is about treatment sessions on parent-child relationships. |
| Integration of the children into the treatment sessions |  | Aspects are coded that are related to the fact that children are actively involved in treatment sessions and how the parents evaluate them, e.g., parent-child swimming or three-way conversations with therapist-child-parent take place. |
| Criticism of parent-related treatment | Therapeutic offers with a child focus | This is about the general criticism of clinics regarding parent-related treatment. e.g., missing age-appropriate toys, the staff that is unfamiliar with the subject, or no meaningful opportunities for children to visit… |
|  | Therapeutic aspects |  |
|  | Structural aspects |  |
|  | Topic school/kindergarten |  |
| Positive aspects of parent-oriented treatment | Topic treatment offers | Code all aspects that are rated positively regarding children or families. Both structural and personal aspects are coded. |
|  | Topic children |  |
|  | Topic therapist (as a person) |  |
|  | Learning through treatment |  |
| Concrete suggestions for improvement |  | This is coded if there are ideas about helpful changes to tailor treatment to parents. |
| Sufficient parenting-related treatment |  | The extent to which the treatment was experienced was sufficient for the parents is coded. |
| Conclusion on treatment |  | General conclusions are coded. e.g., treatment only without an accompanying child, treatment only in another clinic. I feel well prepared for the time after inpatient treatment… |
| Wishes | Structural/therapeutic wishes | Both personal and structural wishes that came up during the interview are openly coded here. |
|  | Personal wishes |  |
| Disease data (including comorbidities) |  | Please code all comorbidities and information on physical and mental diseases that are not SUD. |
