# Supplementary material for: Mapping the subjective importance of the topic ‘parenthood’ for parents with substance use disorder in inpatient rehabilitative care – an explorative qualitative study in Germany
Source: Subst Abuse Treat Prev Policy. 2026 Feb 7;21:21. doi: 10.1186/s13011-026-00707-8 (PMC12977838; doi:10.1186/s13011-026-00707-8)
Supplement: Supplementary file 3 — Supplementary Material 3 [file 13011_2026_707_MOESM3_ESM.docx]

Additional File 3: COREQ Reporting Checklist

| Domain 1: Research team and reflexivity | |
| --- | --- |
| Personal Characteristics | |
| 1. Interviewer/facilitator | Detailed in methods section "Data collection":  Interviewer: Ananda Stullich (AS) |
| 1. Credentials | AS: M.A.  JG: M.A.  JS: M.Sc.PH, M.Sc.  Jana Dehner (JD): B.Sc.  Matthias Richter (MR): Prof. Dr. rer. soc.  Laura Hoffmann (LH): Dr. phil. |
| 1. Occupation | AS, JG, JS, LH: research associates  JD: Student assistant  MR: Professor (Chair of Social Determinants of Health at TUM), project coordinator |
| 1. Gender | AS, LH, JD: Female  JG, JS, MR: Male |
| 1. Experience and training | Interviewers had significant experience in qualitative research and were led by an experienced researcher (MR). Additionally, they attended external workshops and informal training.  Experience:  AS: Significant experience in qualitative research and interviews, realized qualitative interviews  JG: Significant experience in qualitative research and interviews  JD: Experience in qualitative research and interviews  JS: Significant experience in qualitative research and interviews  LH: Significant experience in qualitative research and interviews  MR: conceptualized and realized several qualitative interview studies previously |
| Relationship with participants | |
| 1. Relationship established | The authors who collected the data (AS) or analysed them (AS, LD, LH) had no prior existing relationship with the interviewees. |
| 1. Participant knowledge of the interviewer | Interviewees were informed about the project, interviewer’s educational background and occupational status in advance. Participants had the chance to request further information regarding the provided information. |
| 1. Interviewer Characteristics | The interviewers have a research interest in health services research and (medical) sociology. |
| Domain 2: Study design | |
| Theoretical Framework | |
| 1. Methodological orientation and theory | Reported in the methods section. |
| Participant selection | |
| 1. Sampling | Reported in the methods section. |
| 1. Method of approach | Reported in the methods section. |
| 1. Sample size | Reported in the methods section. |
| 1. Non-participation | Reported in the methods section. |
| Setting | |
| 1. Setting of data collection | Reported in the methods section "Preparing and conducting the interviews":  Interviews: In the clinics. |
| 1. Presence of non-participants | No one else was present besides the participant and the interviewer. |
| 1. Description of Sample | Reported in the methods section. |
| Data Collection | |
| 1. Interview Guideline | The guides are described in the methods section "Data collection and analysis" and can be found in the Additional Files. Follow-up questions were asked individually during the interview. |
| 1. Repeat interviews | No repeat interview was necessary. |
| 1. Audio/Visual recording | Reported in the methods section " Preparing and conducting the interviews ". |
| 1. Field notes | AS wrote research diary and postscripts. No field notes were taken. |
| 1. Duration | Reported in the methods section. The individual interviews lasted between 13 and 70 minutes. |
| 1. Data saturation | Reported in the methods section "Data". |
| 1. Transcripts returned | Transcripts were not returned to participants. |
| Domain 3: Analysis and findings | |
| Data analysis | |
| 1. Number of data coders | Reported in the methods section "Data analysis":  Indexing all interviews and analysing: AS  Index in several double coded interviews: LH, JD  Summarizing and charting of the indexed data: AS, JG, JS, JD, LH |
| 1. Description of the coding tree | Reported in the methods section. See Additional File 2. |
| 1. Derivation of themes | Reported in the methods section "Data analysis". |
| 1. Software | Reported in the methods section "Data analysis". |
| 1. Participant checking | Not reported. |
| Reporting | |
| 1. Quotations presented | Different participants' quotes are presented in the results to illustrate the findings, and a number identifies each quotation. |
| 1. Data and findings consistent | Yes. |
| 1. Clarity of major themes | Major themes are presented in the results. |
| 1. Clarity of minor themes | As far as the word count permits, we discuss minor themes, too. |
